# Supplementary material for: Joint Investigation of 2-Month Post-diagnosis IgG Antibody Levels and Psychological Measures for Assessing Longer Term Multi-Faceted Recovery Among COVID-19 Cases in Northern Cyprus
Source: Front Public Health. 2021 Feb 2;8:590096. doi: 10.3389/fpubh.2020.590096 (PMC7884822; doi:10.3389/fpubh.2020.590096)
Supplement: Supplementary file 1 [file Table_1.DOCX]

**Supplementary Text**

**Blood Collection and Transfer**

Blood samples were taken by trained nurses. Venipuncture was used to collect blood. 10ml complete gel barrier formation tubes were used for blood collection. These vacuum tubes are designed to draw a pre-determined volume of blood. 5-10 ml blood sample was taken for the tests. All sample tubes were labeled anonymously with the patient's assigned study code. Study key list matching study code and participant name was kept by the head-nurse. The collected blood samples were shipped to the central laboratory in temperature-controlled transport boxes. Transport of specimens took place at 18-22°C, and temperature control was maintained by pre-frozen gel packs placed in transport boxes. The samples were transported to the central laboratory and processed within two days. When samples arrived at the lab, centrifuge was used for physically separating the serum from blood cells. The centrifuge was set to spin for 10-15 minutes at 6000 rpm, and each tube was checked immediately to ensure the spinning was effective and their contents have separated. Any remaining serum sample was stored in case the test needed to be repeated.

**Supplementary Results**

The mean score for perceived discrimination due to COVID-19 diagnosis was 2.48 (SD 1.30), borderline mid-level (2.5). Of the 41 survey participants, about half [20 (49%)] reported feeling excluded/discriminated against by family/friends, workplace and/or community because of having COVID-19 infection. Sixteen of these 20 individuals indicated family/friends as one of the sources of exclusion/discrimination. About a third of the survey respondents (13 (32%)) reported feeling differential treatment/communication by the community because of having COVID-19 infection; the perceived differential treatment/communication rate among women and men in was 4 out of 23 (17%) and 9 out of 18 (50%), respectively. Similarly, about a third of the survey respondents (14 (34%)) reported being subjected to verbal harassment or insult due to the COVID-19 infection they had in real life settings and/or on social media (**Supplementary Table 6**).

Interestingly, the mean score for experiencing anxiety as a result of future stigma was below the mid-level at 1.19 (SD=1.06). Nine (22%) and ten (24%) individuals were concerned that their relationship with the workplace and family/friends, respectively, would deteriorate due to the COVID-19 infection they had (**Supplementary Table 7**). We elaborate on this and how it relates to current levels of experiencing the pandemic as a life-changing trauma and negative emotions below.

An assessment of subjective evaluations of health before being tested positive (mean 4.45, SD 0.72) and at the end of the recovery (mean 4.21, SD .83) revealed a slightly worse score for perceived health after infection. Eighty-five percent of respondents assessed their general health prior to COVID-19 infection as good or very good, as compared to 80% post-COVID-19 infection. When ***derived*** change in general health assessment was evaluated, 2% reported much better health, 7% better health, 17% worse health and 5% much worse health for post-COVID assessment as compared to pre-COVID assessment. Fifty-nine percent did not report any change in their general health post-infection. Of the 8 individuals with severe/critical disease, four (50%) did not report any change in their general health, and responses were split evenly among other categories with one (13%) each reporting much better health, better health, worse health, and much worse health for after versus before COVID-19 infection (**Supplementary Table 8**).

Mean score for perceived importance of protective measures (4.40; SD=1.00) was high. Around 80-85% of the respondents thought it was important or very important to follow the recommended/mandatory prevention measures. There was a higher perceived importance of protective measures in individuals with a chronic disease (mean (SD) score of 4.92 (0.29)) compared to those without (mean (SD) score of 4.20 (1.12)) (Wilcoxon rank-sum test; p=0.01). Four to seven percent of responders assessed the stated prevention measures as not or slightly important.

(**Supplementary Table 9**).

Mean score for willingness to help others by sharing information (4.39; SD=0.97) was also high. Thirty-five (85%) respondents mentioned sharing information with loved ones and the community around them to prevent the spread of the infection. (**Supplementary Table 10**).

Among the disease prevention habits, a range of fifty-one to 63% of the survey respondents indicated that they have *always* followed each of the disease prevention habits prior to infection, with survey respondents *always* following *all* four disease prevention habits at 29%. Percent of individuals *never* wearing a mask in the community or observing at least 1-meter distance rule was 15% for each protective measure. Self-reported overall compliance (i.e., *often/always*) with hand-washing (85%), and avoiding close contact (76%) appeared slightly higher than those for mask-wearing (63%) and observing the distance-rule (66%). (**Supplementary Table 11).**

Among the other survey questions, three (7%) respondents believed they could still transmit the virus to someone else. Twenty-five (61%) individuals felt more relieved than before for having had the infection; there was a differential assessment by women (78%) versus men (39%) (p=0.01). Twenty (49%) thought the infection was nothing to be afraid of; two (25%) individuals with severe/critical disease thought the infection was nothing to be afraid of, as compared to 18 (55%) with mild/moderate disease. (**Supplementary Table 12).**
