# Supplementary material for: Joint Investigation of 2-Month Post-diagnosis IgG Antibody Levels and Psychological Measures for Assessing Longer Term Multi-Faceted Recovery Among COVID-19 Cases in Northern Cyprus
Source: Front Public Health. 2021 Feb 2;8:590096. doi: 10.3389/fpubh.2020.590096 (PMC7884822; doi:10.3389/fpubh.2020.590096)
Supplement: Supplementary file 2 [file Table_2.DOCX]

**Supplementary Table 1. Cross-tabulation of Baseline Characteristics and Disease Severity by Age Group**

|  | | **Age Group** | | |
| --- | --- | --- | --- | --- |
|  |  | **0-29 Years (N=9)** | **30-59 Years (N=25)** | **60+ Years (N=13)** |
| **Sex** | Women | 5 (56%) | 14 (56%) | 9 (69%) |
|  | Men | 4 (44%) | 11 (44%) | 4 (31%) |
| **Any Symptom Reported at the Time of Diagnosis** | No | 4 (44%) | 5 (20%) | 1 (8%) |
|  | Yes | 5 (56%) | 20 (80%) | 12 (92%) |
| **Fever/History of Fever Reported at the Time of Diagnosis** | No | 7 (78%) | 9 (36%) | 9 (69%) |
|  | Yes | 2 (22%) | 16 (64%) | 4 (31%) |
| **Comorbidity** | No | 9 (100%) | 18 (72%) | 5 (38%) |
|  | Yes | 0 (0%) | 7 (28%) | 8 (62%) |
| **Disease Severity^1^** | Mild/Moderate | 9 (100%) | 20 (80%) | 9 (69%) |
|  | Severe/Critical | 0 (0%) | 5 (20%) | 4 (31%) |

^1^ Disease severity was defined as critical (requiring intensive care), severe (requiring oxygen therapy, but otherwise stable) and mild/moderate (all other cases including asymptomatic cases).

**Supplementary Table 2** IgG Antibody Titers Overall, by Baseline Characteristics and Disease Severity

|  | | **Median [IQR] IgG Titer (N=47)** | **P-Value** |
| --- | --- | --- | --- |
| **Total** |  | 4.38 [2.05-5.88] | n/a |
| **Sex** | Women (N=28) | 4.30 [2.42-5.78] | **0.94** |
|  | Men (N=19) | 4.51 [1.70-6.08] |  |
| **Age (years)** | 0-29 (N=9) | 3.95 [1.70-4.28] | **0.26** |
|  | 30-59 (N=25) | 4.51 [1.72-6.20] |  |
|  | 60+ (N=13) | 5.52 [3.01-6.08] |  |
| **Any Symptom Reported at the Time of Diagnosis** | No (N=10) | 3.76 [0.27-4.38] | **0.09** |
|  | Yes (N=37) | 4.79 [2.78-6.08] |  |
| **Fever/History of Fever** **Reported** **at the Time of Diagnosis** | No (N=25) | 3.57 [1.47-5.13] | **0.01** |
|  | Yes (N=22) | 5.56 [4.11-6.20] |  |
| **Comorbidity** | No (N=32) | 3.87 [1.25-5.56] | **0.03** |
|  | Yes (N=15) | 5.52 [4.31-6.09] |  |
| **Disease Severity^1^** | Mild/Moderate (N=38) | 3.94 [1.70-5.52] | **0.001** |
|  | Severe/Critical (N=9) | 6.09 [5.88-6.24] |  |

^1^ Disease severity was defined as critical (requiring intensive care), severe (requiring oxygen therapy, but otherwise stable) and mild/moderate (all other cases including asymptomatic cases).

**Supplementary Table 3** IgG Antibody Titers by Baseline Characteristics and Disease Severity

|  |  | **Mild/Moderate (N=38)** | | **Severe/Critical (N=9)** | |
| --- | --- | --- | --- | --- | --- |
|  |  | **N** | **Median [IQR] IgG Titer** | **N** | **Median [IQR] IgG Titer** |
| **Total** |  |  | 3.94 [1.70-5.52] |  | 6.09 [5.88-6.24] |
| **Sex** | Women | 25 | 4.11 [2.05-5.60] | 3 | 5.88 [5.37-7.75] |
|  | Men | 13 | 3.79 [1.47-4.51] | 6 | 6.15 [6.08-6.24] |
| **Age** | 0-29 | 9 | 3.95 [1.70-4.28] | 0 | n/a |
|  | 30-59 | 20 | 4.05 [1.10-5.56] | 5 | 6.24 [6.20-6.97] |
|  | 60+ | 9 | 3.92 [2.78-5.67] | 4 | 5.98 [5.63-6.09] |
| **Any Symptom Reported at the Time of Diagnosis** | No | 10 | 3.76 [0.27-4.38] | 0 | n/a |
|  | Yes | 28 | 4.02 [1.88-5.56] | 9 | 6.09 [5.88-6.24] |
| **Fever/History of Fever** **Reported** **at the Time of Diagnosis** | No | 24 | 3.29 [1.09-5.04] | 1 | 5.37 |
|  | Yes | 14 | 4.41 [3.29-5.58] | 8 | 6.15 [5.98-6.61] |
| **Comorbidity** | No | 28 | 3.43 [0.88-4.87] | 4 | 6.61 [6.06-7.36] |
|  | Yes | 10 | 5.02 [3.92-5.67] | 5 | 6.08 [5.37-6.09] |

**Supplementary Table 4**. Survey Responses for Covid-19 as Life-changing Trauma (CALCT)

|  | **No Response** | **Strongly Disagree** | **Disagree** | **Neither Agree Nor Disagree** | **Agree** | **Strongly Agree** |
| --- | --- | --- | --- | --- | --- | --- |
| 1. In general, I have become a more worried person because of my infection. | 1 (2%) | 13 (32%) | 6 (15%) | 2 (5%) | 9 (22%) | 10 (24%) |
| 2. I perceive this period as a turning point in my life. | 1 (2%) | 8 (20%) | 6 (15%) | 6 (15%) | 5 (12%) | 15 (37%) |
| 3. Due to the Covid-19 infection I had, my perspective on life and my priorities have changed. | 2 (5%) | 9 (22%) | 4 (10%) | 2 (5%) | 13 (32%) | 11 (27%) |

**Supplementary Table 5**. Survey Responses for Negative Emotions

| **How did you feel when you first heard that you were Covid-19 positive?** | **No Response** | **Not at All** | **A Little** | **Moderately** | **A Lot** | **Quite a Lot** |
| --- | --- | --- | --- | --- | --- | --- |
| 1. I felt fear of death | 5 (12%) | 22 (54%) | 1 (2%) | 5 (12%) | 5 (12%) | 3 (7%) |
| 2. I was worried | 2 (5%) | 7 (17%) | 3 (7%) | 11 (27%) | 2 (5%) | 16 (39%) |
| 3. I felt helpless | 4 (10%) | 15 (37%) | 3 (7%) | 4 (10%) | 4 (10%) | 11 (27%) |
| 4. I felt guilty because I was not sufficiently protected | 3 (7%) | 25 (61%) | 5 (12%) | 5 (12%) | 1 (2%) | 2 (5%) |

**Supplementary Table 6**. Survey Responses for Perceived Discrimination

|  | **No Response** | **Strongly Disagree** | **Disagree** | **Neither Agree Nor Disagree** | **Agree** | **Strongly Agree** |
| --- | --- | --- | --- | --- | --- | --- |
| 1. I feel excluded/discriminated by my family/friends due to the Covid-19 infection I had. | 2 (5%) | 15 (37%) | 5 (12%) | 3 (7%) | 5 (12%) | 11 (27%) |
| 2. I feel excluded/discriminated by my workplace because of the Covid-19 infection I had. | 6 (15%) | 18 (44%) | 5 (12%) | 3 (7%) | 4 (10%) | 5 (12%) |
| 3. I feel that I have been excluded/discriminated by the community because of my Covid-19 infection. | 3 (7%) | 10 (24%) | 8 (20%) | 4 (10%) | 4 (10%) | 12 (29%) |
| 4. I feel that people treat me, speak to me differently due to the Covid-19 infection I had. | 1 (2%) | 13 (32%) | 9 (22%) | 5 (12%) | 4 (10%) | 9 (22%) |
| 5. I was subjected to verbal harassment or insult due to the Covid-19 infection I had. | 1 (2%) | 24 (59%) | 6 (15%) | 0 (0%) | 4 (10%) | 6 (15%) |
| 6. I was subjected to verbal harassment or insult on social media due to the Covid-19 infection I had. | 1 (2%) | 24 (59%) | 3 (7%) | 1 (2%) | 5 (12%) | 7 (17%) |

**Supplementary Table 7**. Survey Responses for Future Stigma Related Anxiety (FSA)

|  | **No Response** | **Strongly Disagree** | **Disagree** | **Neither Agree Nor Disagree** | **Agree** | **Strongly Agree** |
| --- | --- | --- | --- | --- | --- | --- |
| 1. I am concerned that my work, my relationship with the workplace will deteriorate due to the Covid-19 infection I had. | 4 (10%) | 21 (51%) | 2 (5%) | 5 (12%) | 5 (12%) | 4 (10%) |
| 2. I am concerned that my relationship with my family and/or friends will deteriorate due to the Covid-19 infection I had. | 1 (2%) | 24 (59%) | 5 (12%) | 1 (2%) | 7 (17%) | 3 (7%) |

**Supplementary Table 8.** Survey Responses for Perception of Global Health Before and After Diagnosis

|  | **No Response** | **Very Poor** | **Poor** | **Fair** | **Good** | **Very Good** |
| --- | --- | --- | --- | --- | --- | --- |
| 1. In general, how would you rate your health prior to Covid-19 infection? | 3 (7%) | 0 (0%) | 1 (2%) | 2 (5%) | 14 (34%) | 21 (51%) |
| 2. In general, how would you rate your health post Covid-19 infection (i.e., now)? | 2 (5%) | 0 (0%) | 2 (5%) | 4 (10%) | 17 (41%) | 16 (39%) |
|  | **No Response^1^** | **Much Worse^2^** | **Worse** | **Same** | **Better** | **Much Better^3^** |
| ***Derived*** change in general health assessment after Covid-19 infection (question 2) as compared to before (question 1) | 4 (10%) | 2 (5%) | 7 (17%) | 24 (59%) | 3 (7%) | 1 (2 %) |

^1^ No response to either question

^2^ Change down two levels or more.

^3^ Change up two levels or more.

**Supplementary Table 9.** Survey Responses for Perceived Importance of Protective Measures

| **How important do you think the following measures are to prevent the spread of the outbreak?** | **No Response** | **Not Important** | **Slightly Important** | **Indecisive** | **Important** | **Very Important** |
| --- | --- | --- | --- | --- | --- | --- |
| 1. Frequent and detailed hand washing | 2 (5%) | 1 (2%) | 1 (2%) | 3 (7%) | 8 (20%) | 26 (63%) |
| 2. Avoiding close contact such as handshaking, kissing, hugging in the community | 2 (5%) | 2 (5%) | 0 (0%) | 2 (5%) | 9 (22%) | 26 (63%) |
| 3. Observing at least 1 meter distance rule | 2 (5%) | 3 (7%) | 0 (0%) | 2 (5%) | 9 (22%) | 25 (61%) |
| 4. Wearing a mask in the community | 3 (7%) | 3 (7%) | 0 (0%) | 3 (7%) | 5 (12%) | 27 (66%) |

**Supplementary Table 10.** Survey Responses for Pro-social tendencies

|  | **No Response** | **Strongly Disagree** | **Disagree** | **Neither Agree Nor Disagree** | **Agree** | **Strongly Agree** |
| --- | --- | --- | --- | --- | --- | --- |
| 1. I share information with my loved ones and the people around me to protect them from infection. | 3 (7%) | 2 (5%) | 0 (0%) | 1 (2%) | 13 (32%) | 22 (54%) |

**Supplementary Table 11** Survey Responses for Disease Prevention Habits

| **How much did you follow the general precautions against coronavirus (Covid-19) before infection?** | **No Response** | **Never** | **Rarely** | **Sometimes** | **Often** | **Always** |
| --- | --- | --- | --- | --- | --- | --- |
| 1. Frequent and detailed hand washing | 1 (2%) | 2 (5%) | 0 (0%) | 3 (7%) | 9 (22%) | 26 (63%) |
| 2. Avoiding close contact such as handshaking, kissing, hugging in the community | 1 (2%) | 1 (2%) | 2 (5%) | 6 (15%) | 7 (17%) | 24 (59%) |
| 3. Observing at least 1 meter distance rule | 3 (7%) | 6 (15%) | 0 (0%) | 6 (15%) | 5 (12%) | 21 (51%) |
| 4. Wearing a mask in the community | 2 (5%) | 6 (15%) | 1 (2%) | 5 (12%) | 5 (12%) | 22 (54%) |

**Supplementary Table 12** Other Survey Questions and Responses

|  | **No Response** | **Strongly Disagree** | **Disagree** | **Neither Agree Nor Disagree** | **Agree** | **Strongly Agree** |
| --- | --- | --- | --- | --- | --- | --- |
| 1. I believe I can transmit to virus to someone else now. | 1 (2%) | 22 (54%) | 9 (22%) | 6 (15%) | 0 (0%) | 3 (7%) |
| 2. I feel more relieved than before because I had the infection. | 3 (7%) | 4 (10%) | 3 (7%) | 6 (15%) | 12 (29%) | 13 (32%) |
| 3. I think the infection is nothing to be afraid of. | 2 (5%) | 11 (27%) | 6 (15%) | 2 (5%) | 8 (20%) | 12 (29%) |
