# Supplementary material for: Joint Investigation of 2-Month Post-diagnosis IgG Antibody Levels and Psychological Measures for Assessing Longer Term Multi-Faceted Recovery Among COVID-19 Cases in Northern Cyprus
Source: Front Public Health. 2021 Feb 2;8:590096. doi: 10.3389/fpubh.2020.590096 (PMC7884822; doi:10.3389/fpubh.2020.590096)
Supplement: Supplementary file 3 [file Table_3.DOCX]

**COVID-19 POST-DISCHARGE INFORMATION FORM AND SURVEY**

Sequence No: Form Completion Date:

Address:..................................................................................................................................

Name:................................................... Date of Birth:.......................................................

Cell Phone No:..........**............................**.......................

Education Status: .............................................................

Occupation: 1- ……………………………………….. 2- Retired

**1-Total number of people living at your home:**

1-Under 20 years …………………………........ people

2-20 years or older…………………………........ people

**2-Do you have a chronic disease?**

1- No 2- Cardiovascular Disease 3- COPD 4- Diabetes 5- Hypertension

6- Cancer 7- Asthma 8- Other……………….......................................................

Indicate any medications taken for these diseases: …….............…………………………..

**In the questions below, mark ‘X’ in the box that most closely reflects your opinion.**

**3-**

| **How much did you follow the general precautions against coronavirus (Covid-19) before infection?** | **Never** | **Rarely** | **Sometimes** | **Often** | **Always** |
| --- | --- | --- | --- | --- | --- |
| 1. Frequent and detailed hand washing |  |  |  |  |  |
| 2. Avoiding close contact such as handshaking, kissing, hugging in the community |  |  |  |  |  |
| 3. Observing at least 1 meter distance rule |  |  |  |  |  |
| 4. Wearing a mask in the community |  |  |  |  |  |

**4-**

| **How important do you think the following measures are to prevent the spread of the outbreak?** | **Not Important** | **Slightly Important** | **Indecisive** | **Important** | **Very Important** |
| --- | --- | --- | --- | --- | --- |
| 1. Frequent and detailed hand washing |  |  |  |  |  |
| 2. Avoiding close contact such as handshaking, kissing, hugging in the community |  |  |  |  |  |
| 3. Observing at least 1 meter distance rule |  |  |  |  |  |
| 4. Wearing a mask in the community |  |  |  |  |  |

**5-**

| **How did you feel when you first heard that you were Covid-19 positive?** | **Not at All** | **A Little** | **Moderately** | **A Lot** | **Quite a Lot** |
| --- | --- | --- | --- | --- | --- |
| 1. I felt fear of death |  |  |  |  |  |
| 2. I was worried |  |  |  |  |  |
| 3. I felt anger |  |  |  |  |  |
| 4. I felt helpless |  |  |  |  |  |
| 5. I felt guilty because I was not sufficiently protected |  |  |  |  |  |

**6-**

|  | **Very Poor** | **Poor** | **Fair** | **Good** | **Very Good** |
| --- | --- | --- | --- | --- | --- |
| 1. In general, how would you rate your health prior to Covid-19 infection? |  |  |  |  |  |
| 2. In general, how would you rate your health post Covid-19 infection (i.e., now)? |  |  |  |  |  |
|  | **Very Poor** | **Poor** | **Same** | **Good** | **Very Good** |
| 3. How would you compare your general health prior to Covid-19 diagnosis with post Covid-19 infection (i.e., now)? |  |  |  |  |  |

**7-**

|  | **Strongly Disagree** | **Disagree** | **Neither Agree Nor Disagree** | **Agree** | **Strongly Agree** |
| --- | --- | --- | --- | --- | --- |
| 1. I feel excluded/discriminated by my family/friends due to the Covid-19 infection I had. |  |  |  |  |  |
| 2. I feel excluded/discriminated by my workplace because of the Covid-19 infection I had. |  |  |  |  |  |
| 3. I feel that I have been excluded/discriminated by the community because of my Covid-19 infection. |  |  |  |  |  |
| 4. I feel that people treat me, speak to me differently due to the Covid-19 infection I had. |  |  |  |  |  |
| 5. I was subjected to verbal harassment or insult due to the Covid-19 infection I had. |  |  |  |  |  |
| 6. I was subjected to verbal harassment or insult on social media due to the Covid-19 infection I had. |  |  |  |  |  |

**8-**

|  | **Strongly Disagree** | **Disagree** | **Neither Agree Nor Disagree** | **Agree** | **Strongly Agree** |
| --- | --- | --- | --- | --- | --- |
| 1. In general, I have become a more worried person because of my infection. |  |  |  |  |  |
| 2. I perceive this period as a turning point in my life. |  |  |  |  |  |
| 3. I believe I can transmit to virus to someone else now. |  |  |  |  |  |
| 4. I am concerned that my work, my relationship with the workplace will deteriorate due to the Covid-19 infection I had. |  |  |  |  |  |
| 5. I am concerned that my relationship with my family and/or friends will deteriorate due to the Covid-19 infection I had. |  |  |  |  |  |

**9-**

|  | **Strongly Disagree** | **Disagree** | **Neither Agree Nor Disagree** | **Agree** | **Strongly Agree** |
| --- | --- | --- | --- | --- | --- |
| 1. Due to the Covid-19 infection I had, my perspective on life and my priorities have changed. |  |  |  |  |  |
| 2. I feel more relieved than before because I had the infection. |  |  |  |  |  |
| 3. I think the infection is nothing to be afraid of. |  |  |  |  |  |
| 4. I share information with my loved ones and the people around me to protect them from infection. |  |  |  |  |  |
